# Estimating the number and size of the main effects in genome-wide case-control association studies

**DOI:** 10.1186/1753-6561-1-s1-s143

**Published:** 2007-12-18

**Authors:** Po-Hsiu Kuo, József Bukszár, Edwin JCG van den Oord

**Affiliations:** 1 Virginia Institute for Psychiatric and Behavioral Genetics, Virginia Commonwealth University, 800 East Leigh Street, Biotech 1, VIPBG, Suite 1-130, Richmond, Virginia 23219, USA; 2 Institute of Clinical Medicine, College of Medicine, National Cheng Kung University, 138, Sheng-Li Road, Tainan 704, Taiwan; 3 Center for Biomarker Research and Personalized Medicine, Virginia Commonwealth University, 410 North 12th Street, R Blackwell Smith Building, Richmond, Virginia 23219, USA

## Abstract

It has recently become possible to screen thousands of markers to detect genetic causes of common diseases. Along with this potential comes analytical challenges, and it is important to develop new statistical tools to identify markers with causal effects and accurately estimate their effect sizes. Knowledge of the proportion of markers without true effects (*p*_0_) and the effect sizes of markers with effects provides information to control for false discoveries and to design follow-up studies. We apply newly developed methods to simulated Genetic Analysis Workshop 15 genome-wide case-control data sets, including a maximum likelihood (ML) and a quasi-ML (QML) approach that incorporate the test statistic distribution and estimates effect size simultaneously with *p*_0_, and two conservative estimators of *p*_0 _that do not rely on the test statistic distribution under the alternative. Compared with four existing commonly used estimators for *p*_0_, our results illustrated that all of our estimators have favorable properties in terms of the standard deviation with which *p*_0 _is estimated. On average, the ML method performed slightly better than the QML method; the conservative method performed well and was even slightly more precise than the ML estimators, and can be more robust in less optimal conditions (small sample sizes and small number of markers). Further improvements and extensions of the proposed methods are conceivable, such as estimating the distribution of effect sizes and taking population stratification into account when obtain estimates of *p*_0 _and effect size.

## Background

Due to the rapid advances in genotyping technology, genome-wide association studies with hundreds of thousands of markers are now possible. These large scale genetic studies offer great promise to expedite the discovery of the common genetic variants affecting common diseases [1]. A first step in the analyses is to understand the properties of the massive data sets. Among the most fundamental properties are the proportion of markers without true effects (*p*_0_) and the effect sizes (Δ) of markers with effects. Knowledge of these parameters provides information about how relevant the genotyped markers are for the disease outcome. In addition, these parameters play a role in a variety of applications. For example, estimates of *p*_0 _are commonly used in methods for controlling false discoveries, which is important to prevent spending time and resources on leads that will eventually prove irrelevant. Another example is that knowledge of effect size Δ is important to design follow-up studies that have adequate power to replicate previous findings.

Multiple methods have been proposed to estimate *p*_0 _[2-4]. These estimators tend to make general assumptions about the distribution of the test statistic under the alternative hypothesis, partly because many of them have been developed in the context of microarray research where specific assumptions may be problematic. However, in genetics good approximations for the statistical test statistic distribution are often available. This information can be used to obtain more precise estimators in these applications. Once (a set of) markers have been identified as being associated with the disease, the next objective of interest is typically to estimate the effect sizes. Currently, the most commonly used approach simply estimates the effect size of the significant markers in the same sample that has been used for testing. Due to the effects of sampling error and the presence of false positives, this approach overestimates the effect sizes considerably [5,6]. Several methods have been proposed to obtain more unbiased effect size estimates such as a simple split-sample method, cross-validation, and bootstrap resampling [7]. However, with all these methods that use the same sample to first declare significance and then estimate the effects sizes of the significant finding, it will remain difficult to obtain estimates that are both precise and unbiased. We therefore proposed a set of related methods (unpublished data) that estimate the average effect size Δ of the 1 - *p*_0 _markers with effects. Because our estimates are not confined to only those markers that are declared significant, they do not suffer from the upward bias caused by sampling fluctuations producing large test statistics in this specific sample. Our methods do not assign effect size estimates to individual markers but estimate the average of all markers with effects. This does not hamper the design of subsequent replication studies. Thus, for any critical value chosen to declare significance, we can calculate the number of markers with effects plus their average effect size among the significant markers to design replication studies.

Our methods include a maximum likelihood (ML) and a quasi-ML (QML) approach that incorporates the test statistic distribution and estimates Δ simultaneously with *p*_0_. In addition, we propose a conservative estimators of *p*_0 _(CON) and a variation of this conservative estimator that adaptively estimates a fine tuning parameter (ADA CON). Neither CON nor ADA CON rely on the test statistic distribution under the alternative but take advantage of the specific knowledge that in large-scale genetic studies the *p*_0 _must be very close to 1. Because these conservative estimators do not consider the distribution under the alternative hypothesis, they cannot estimate the average effect size directly. However, we can still use the point estimate of these conservative methods and include it in a second step in a ML method to estimate the average effect size Δ for our conservative estimators of *p*_0_. We apply our methods to the simulated rheumatoid arthritis (RA) case-control data with 10 k single-nucleotide polymorphisms (SNPs) in Genetic Analysis Workshop 15 (GAW15). We chose a case-control design with SNPs because this is one of the most important designs for mapping the genetic determinants of complex human diseases through genome-wide association studies. We also compared our estimators with four existing estimators. We found two studies comparing multiple and non-overlapping sets of estimators [2,3]. In these studies, the lowest slope (LOW S) and location based estimator (LBE) showed the most favorable properties and were therefore included here. In addition, estimators developed by Storey (STO) [4] and Storey-Tibshirani (STO-TIB) [8] were included because they may be among the more commonly used estimators.

## Methods

The maximum likelihood methods (ML and QML) and the conservative methods (CON and ADA CON) are briefly described below. All 100 replicates in GAW15 Problem 3 were analyzed. To create a case-control data set we selected the first sib from family-based data sets as independent cases (*N *= 1500), and used all individuals in control data sets.

Analyses were done with knowledge of the "answers" of causal markers locations.

### A single-value approximation for Pearson's statistic

SNPs are bi-allelic so that the initial statistical analysis will consist of calculating Pearson's statistic to test whether the frequency of the two alleles (A, a) or three SNP genotypes (AA, Aa, aa) differs between cases and controls. For Pearson's test we can define a single parameter Δ that be interpreted as an (average) effect size. For 2 × 2 tables, for example,

$$\Delta =\frac{\sqrt{\gamma \delta }\sqrt{q_{1}\left(1-q_{1}\right)}\left(o-1\right)}{\sqrt{\left(\left(o-1\right)\left(\gamma +\delta q_{1}\right)+1\right)\left(\left(o-1\right)\delta q_{1}+1\right)}}.$$

where *o *is the odds ratio, *γ *and *δ *= 1 - *γ *the proportions of controls and cases, *q*_1 _and 1 - *q*_1 _the allele frequency in the controls and cases.

We can derive the following approximation [9] for the distribution of Pearson's statistic to analyze for 2 × *ν *contingency tables that depends on only Δ.

$$\chi_{\nu -2}+\left(1-\Delta^{2}\right)\chi_{1}\left(\frac{n\Delta^{2}}{1-\Delta^{2}}\right),$$

where *χ*_*ν*-2 _is a (central) chi-square random variable with *ν *- 2 degrees of freedom and $\chi_{1}\left(\frac{n\Delta^{2}}{1-\Delta^{2}}\right)$ is a chi-square random variable with 1 degree of freedom and non-centrality parameter $\frac{n\Delta^{2}}{1-\Delta^{2}}$. The fact that an approximation exist that depends on only a single parameter (this does not have to be the case as the asymptotic equivalent depends on many parameters) is of great importance because it means that we only have to estimate a single parameter from the data the characterize the effect size. Note that if Δ = 0, the approximation reduces to a central chi-square random variable with *ν *- 1 degrees of freedom under the null hypothesis. In classic works on power analysis [10], categorical data analysis [11], and text books [12], the distribution of Pearson's statistic is often approximated with a non-central chi-square distribution with *ν *- 1 degrees of freedom and non-centrality parameter *n*Δ^2^, which also depends on the single value Δ only. However, this approximation can be inaccurate [9].

### The maximum likelihood estimators

The likelihood function on the *m *test statistics *t*_1_,...,*t*_m _is

$$L(m_{1},\Delta )=\frac{1}{\left(\begin{array}{c} m\\ m_{1} \end{array}\right)}\left(\prod_{i=1}^{m}f_{0}(t_{i})\right)\sum_{\left\{i_{1},...,i_{m_{1}}\}\in \{1,...,m\right\}}\frac{f_{\Delta }\left(t_{i_{1}}\right)}{f_{0}\left(t_{i_{1}}\right)}\times ...\times \frac{f_{\Delta }\left(t_{i_{m_{1}}}\right)}{f_{0}\left(t_{i_{m_{1}}}\right)},$$

where *m*_1 _= *m *- *m*_0 _the number of effects and *m*_0 _the number of markers without effect, *f*_0 _an approximating density function under the null, and *f*Δ an approximating density function under the alternative that depends on average effect size Δ. The ML estimator of *m*_1 _and the average effect size are the ${\hat{m}}_{1}$ and $\overset{⌢}{\Delta }$ that maximize function *L*.

Due to enormous number of terms in the sum, the likelihood cannot be evaluated directly. For example, with a total number of tests *m *= 100,000, of which *m*_1 _= 5 markers have an effect, there are 8.33 × 10^22 ^terms. Therefore, we developed an implementation that uses recursive series to calculate the likelihood. In addition, we developed a quasi-likelihood approach (QML) that is computationally much easier and faster. Here the logarithm on the *m *test statistics *t*_1_,...,*t*_m _is

$$\ell_{quasi}(p_{0},\Delta )=\sum_{i=1}^{m}\log\{p_{0}f_{0}(t_{i})+(1-p_{0})f_{\Delta }(t_{i})\},$$

which is essentially the log-likelihood function of the mixture model.

### The conservative estimator

In addition to the ML estimator, we propose an estimate of *p*_0 _that does not rely on the test statistic distribution under the alternative but capitalizes on the knowledge that in large-scale genetic studies *p*_0 _is close to 1 (CON method). We calculate a cut-off value *c *in such a way that the probability that a non-causal marker has test statistic value higher than *c *is *k*/*m*. If we denote the total number of markers whose test statistic value is higher than *c *as *d*, then this estimate of *p*_0 _is

$${\overset{⌢}{p}}_{0}=1-\frac{d-k}{m}.$$

Note that the expected number of non-causal markers with test statistic value higher than the cut off c is *km*_0_/*m *rather than *k*. This estimator can therefore be expected to be conservatively biased. However, because *p*_0 _= *m*_0_/*m *is close to 1, we would expect the bias to be small.

A natural idea is to choose a value for fine-tuning parameter *k *that minimizes the mean square error $MSE\left(k\right)=E{\left({\overset{⌢}{p}}_{0}-p_{0}\right)}^{2}$ for which an analytical expression can be derived (not shown). A practical problem is that the value of *k *that minimizes the MSE depends on the unknown parameters *p*_0_, the average effect size, and the covariances among the markers. Alternatively, we can estimate *k *from the data (ADA CON method). That is, we first estimate *p*_0 _for a chosen value of *k*, e.g., *k *= 10. Second, using that point estimate, we obtain an estimate of the average effect size (e.g., by ML). Third, for the *p*_0 _and the effect size estimate, we calculate the optimal *k*. We repeat Steps 1 to 3 until there is no noticeable change in *k*. However, extensive simulation showed that this resulted in somewhat less precise estimates than just calculating a value of *k *using reasonable assumptions. The reason was that the conservative method appeared fairly robust against mis-specifications of *k*, which outweighed the additional sampling error associated with estimating *k*.

## Results

We identified four markers on chromosome 6 with extremely low *p*-values and effect sizes that were five times larger than the average effect sizes of the other markers with effects (see Table 1). Because a complex statistical method is not needed to detect such effects, we excluded these four markers and analyzed the remaining set of markers (*N *= 9183). Table 1 displays results across the 100 replicates. Whereas our estimators and LOW S never estimated *p*_0 _to be 1, LBE consistently estimated *p*_0 _to be 1, and STO and STO-TIB were somewhere in between. The mean *p*_0 _estimates were very close to each other in our four new methods but deviated from four existing methods. The only exception was the LOW S method, in which the mean *p*_0 _estimate was closer to what we obtained from the new methods. The precision of *p*_0 _estimates was also high in the new methods as the standard deviations were small.

**Table 1:** Estimating *p*_0 _and average effect size with different methods using all 100 replicates

	*p*_0 _Estimates			Average effect size estimates	
		
	No. times *p*_0 _= 1	Mean	Std. dev.	Mean	Std. dev.
Quasi-maximum likelihood	0	0.998425	1.97 × 10^-4^	0.083	5.10 × 10^-3^
Maximum likelihood	0	0.998424	1.92 × 10^-4^	0.083	4.92 × 10^-3^
Conservative (k = 1)	0	0.998409	1.72 × 10^-4^	0.083	4.94 × 10^-3^
Adaptive conservative	0	0.998425	2.12 × 10^-4^	0.083	5.09 × 10^-3^
Lowest slope	0	0.998162	2.99 × 10^-4^	0.082	5.09 × 10^-3^
Storey-Tibshirani	38	0.984106	1.87 × 10^-2^	0.052	2.79 × 10^-2^
Storey^a^	2	0.972607	2.59 × 10^-2^	0.051	2.75 × 10^-2^
Location based estimator	100	1	0	---	---

^a^For Storey's, estimator we used the grid he suggested in his article (0.01, 0.02,...,0.95).

^b^---, *p*_0 _= 1. There is no marker to estimate effect size.

Based on the *p*_0 _estimate in our new methods, the average number of total causal markers with main effects was 18. The average numbers of causal markers in the LOW S, STO-TIB, STO, and LBE were 21, 150, 256, and 0, respectively. Clearly, STO-TIB and STO overestimated the number of effects and LBE underestimated the number of effects. It is also important to note that standard errors of the estimates were about 100 times larger for STO-TIB and STO, implying that the number of markers was estimated very imprecisely.

The second part of Table 1 shows results for the estimated average effect size Δ. The ML methods estimate Δ and *p*_0 _simultaneously. The other estimators do not consider the distribution under the alternative hypothesis, and can therefore not estimate the average effect size directly. However, in these cases we can still use the point estimate $p_{0}^{*}$ obtained with these estimators and include that in the a maximum likelihood method that finds $\overset{⌢}{\Delta }$ by maximizing ℓ(*m *- *m*$p_{0}^{*}$, Δ). In cases where the point estimate $p_{0}^{*}$ equals 1, the effect size cannot be estimated. In these scenarios Δ was treated as "missing". Results showed that the estimated average effect size was 0.083 in all four new methods in which the ML method was slightly more precise. The estimated average effect size was less precise and considerably lower with STO and STO-TIB, reflecting the downward bias and larger standard deviation in these *p*_0 _estimates.

## Discussion

Results illustrated that all of our four new estimators have favorable properties in terms of the standard deviation with which *p*_0 _is estimated. The ML and QML estimators have the additional advantage that they provide a direct estimate of average effect size Δ. Because the point estimates of *p*_0 _in both CON and ADA CON methods are very similar to that in the ML and QML methods, the average effect size is expected to be similar across methods. This is important because these two parameters are somewhat intertwined and the estimate of the average effect size helps the interpretation of the *p*_0 _estimate. For example, without this effect size estimate, it is unclear whether the estimated numbers of causal markers have very small or large effects.

On average, the ML method performed slightly better than the QML method. Furthermore, we found in other simulations that the QML estimator can be unstable. In general, the ML method may therefore be the method of choice. Results also showed that the CON method performed well and was even slightly more precise than the ML estimators. One reason is that the CON method only estimates a single parameter, whereas the ML methods estimate two parameters. However, this observation is also consistent with previous simulations showing that in less optimal conditions (small sample sizes and small number of markers), the CON method can be more robust. Indeed, as another example of its relative robustness, the CON method performed equally well when the four markers with extremely large effects were included but the ML estimators became somewhat less precise.

Linkage disequilibrium causes test statistics between markers to be correlated. Extensive simulations were performed to examine the impact of such correlated tests on our estimates of the *p*_0 _and Δ (data not shown). Results demonstrated that correlated tests mainly increase the variance of these estimates but did not introduce bias. This makes intuitive sense and essentially mimics other scenarios where certain statistics (e.g., mean) are estimated with correlated observations.

Further improvements and extensions of the proposed methods are conceivable. An example involves work we are currently doing to estimate the distribution of effect sizes. The extension essentially consists of conditioning on the number of markers with effects and then maximizing the likelihood L(Δ|*m*_1_). Thus, we start with estimating the largest effect in the data set, then the second largest, continuing until the estimated effect sizes become (very) small. Another example is that in case-control studies, population stratification can cause spurious associations between marker alleles and disease status when both disease prevalence and allele frequencies differ among subgroups. Using the principle of genomic control [1,13,14], our estimators can be further adapted to obtain estimates of *p*_0 _and Δ that take stratification into account.

## Competing interests

The author(s) declare that they have no competing interests.
